# Clinical effects of Sizi Powder external application combined with TDP therapy for upper limb lymphedema after breast cancer surgery: A retrospective cohort study

**DOI:** 10.1097/MD.0000000000050648

**Published:** 2026-10-02

**Authors:** Juan Zeng, Liwen Xu, Yi Zhang, Huili Xu

**Affiliations:** a Department of Oncology, The Central Hospital of Wuhan, Tongji Medical College, Huazhong University of Science and Technology, Wu’han, Hubei, China; b Key Laboratory for Molecular Diagnosis of Hubei Province, The Central Hospital of Wuhan, Tongji Medical College, Huazhong University of Science and Technology, Wuhan, China; c Department of Nursing, The Central Hospital of Wuhan, Tongji Medical College, Huazhong University of Science and Technology, Wu’han, China; d Department of Surgery, The Central Hospital of Wuhan, Tongji Medical College, Huazhong University of Science and Technology, Wu’han, Hubei, China.

**Keywords:** breast cancer, rehabilitation, retrospective cohort study, Sizi Powder, TDP therapy, upper limb lymphedema

## Abstract

Upper limb lymphedema is a common complication after breast cancer surgery and may substantially impair upper limb function and quality of life. In clinical rehabilitation practice, external application of Sizi Powder combined with TDP therapy has been used as an adjunctive intervention; however, the available clinical evidence remains limited. This study aimed to evaluate the efficacy and safety of Sizi Powder external application combined with TDP therapy in patients with upper limb lymphedema after breast cancer surgery. This single-center retrospective cohort study included 86 patients with unilateral upper limb lymphedema after breast cancer surgery. According to the treatment received in routine clinical practice, patients were assigned to either a combination group treated with Sizi Powder external application plus TDP therapy (n = 44) or a control group receiving conventional rehabilitation management alone (n = 42). Outcome measures included changes in arm circumference difference, symptom scores, upper limb functional indicators, overall treatment response, and treatment-related adverse events. Multivariable logistic regression analysis was further performed to identify factors associated with clinical improvement. Both groups showed improvement after treatment, whereas the combination group demonstrated greater reductions in arm circumference difference at the wrist, 10 cm below the elbow, the elbow, and 10 cm above the elbow. Posttreatment swelling, pain, and heaviness scores were significantly lower in the combination group than in the control group. In addition, shoulder flexion, shoulder abduction, and DASH score improved more markedly in the combination group. Although the overall response rate was higher in the combination group than in the control group, the between-group difference did not reach statistical significance. Adverse events were mild and infrequent in both groups, and no serious treatment-related adverse events were observed. After adjustment for selected baseline covariates, combination treatment remained significantly associated with clinical improvement. Sizi Powder external application combined with TDP therapy may be a safe and beneficial adjunctive intervention for patients with upper limb lymphedema after breast cancer surgery, particularly in reducing swelling, alleviating symptom burden, and improving selected functional outcomes. Larger prospective studies are warranted to further confirm these findings.

## 1. Introduction

Breast cancer is the most commonly diagnosed malignancy among women worldwide and remains a major public health concern.^[1]^ With continued advances in early detection, surgical techniques, systemic therapy, and radiotherapy, survival outcomes have improved substantially over recent decades. Accordingly, the focus of clinical care has gradually expanded from tumor control alone to long-term recovery, functional rehabilitation, and quality of life after treatment.^[2]^ In this context, treatment-related complications have attracted increasing attention, particularly those that persist after surgery and interfere with daily functioning. Among these, upper limb lymphedema is one of the most frequent and clinically significant sequelae after breast cancer treatment.^[3]^

Breast cancer-related lymphedema (BCRL) generally develops as a consequence of impaired lymphatic drainage following surgery or radiotherapy. Axillary lymph node dissection, sentinel lymph node biopsy, postoperative scar formation, and radiation-induced fibrosis may all contribute to lymphatic obstruction or disruption of normal lymphatic flow.^[4]^ Long-term follow-up studies have shown that the risk of lymphedema may continue to accumulate over time after treatment, especially in patients with greater axillary treatment burden, radiotherapy exposure, obesity, or postoperative complications.^[5]^ Clinically, patients may present not only with visible enlargement of the arm, but also with heaviness, pain, tightness, numbness, stiffness, and restricted shoulder mobility. In more severe or persistent cases, tissue fibrosis, recurrent skin infection, and progressive functional impairment may occur.^[6]^ Therefore, upper limb lymphedema should not be regarded as a minor postoperative complaint, but rather as an important issue in rehabilitation and long-term survivorship care.

The burden of lymphedema extends beyond local physical symptoms. Persistent swelling and impaired upper limb function may interfere with dressing, lifting, household tasks, occupational activity, and sleep. Many patients also report emotional distress, reduced self-image, frustration, and a prolonged sense of physical limitation. Previous studies have shown that BCRL is associated with reduced physical, psychological, and social well-being, and that its adverse effects may persist for years after completion of cancer treatment.^[7]^ For this reason, effective prevention and management of postoperative lymphedema have become important components of comprehensive breast cancer rehabilitation.

At present, conservative treatment remains the mainstay of management for upper limb lymphedema after breast cancer surgery. Common approaches include compression therapy, manual lymphatic drainage, exercise rehabilitation, skin care, and complete decongestive therapy. These interventions are widely recommended and play an important role in routine clinical practice. However, their therapeutic effects are not always satisfactory in all patients. Some individuals experience slow improvement, incomplete symptom relief, or poor adherence because treatment may be inconvenient, uncomfortable, costly, or time-consuming.^[7]^ In addition, patients with chronic or longstanding edema often respond less favorably to conventional rehabilitation alone. These limitations have prompted continued interest in adjunctive interventions that are feasible, well tolerated, and potentially capable of improving symptom control and functional recovery.

Recent studies suggest that some conventional adjunctive approaches may offer benefit, although the overall evidence remains heterogeneous. A systematic review and meta-analysis indicated that manual lymphatic drainage may be helpful in selected patients with BCRL, but its effectiveness remains inconsistent across studies.^[8]^ Exercise-based rehabilitation, including resistance exercise, appears to be safe for most breast cancer survivors and may improve physical function without worsening lymphedema.^[9]^ Taken together, these findings support the value of conservative rehabilitation while also highlighting the need to explore additional complementary approaches that may further improve symptom relief and functional outcomes.^[10]^

In Chinese rehabilitation practice, external application of traditional herbal medicine has long been used as an adjunctive treatment for postoperative swelling, pain, and soft tissue discomfort. Sizi Powder is one of the herbal formulations commonly used for this purpose. Although its exact mechanism of action has not yet been fully clarified, it has been applied clinically with the aim of alleviating local swelling and discomfort. TDP therapy, a form of therapeutic electromagnetic spectrum irradiation, is also widely used in rehabilitation medicine. In routine clinical settings, it is believed to promote local circulation and tissue metabolism through thermal and physical effects. Because these 2 methods are often used together, they may provide complementary benefits in patients with edema-related symptoms, particularly during postoperative rehabilitation. However, evidence supporting the combination of Sizi Powder external application and TDP therapy for breast cancer-related upper limb lymphedema remains limited.

Therefore, the present retrospective cohort study was conducted to investigate the efficacy and safety of Sizi Powder external application combined with TDP therapy in patients with upper limb lymphedema after breast cancer surgery. By comparing patients who received the combined intervention with those who received conventional rehabilitation management alone, we aimed to provide preliminary clinical evidence regarding its potential role in postoperative rehabilitation.

## 2. Materials and methods

### 2.1. Study design

This was a single-center retrospective cohort study conducted at Wuhan Central Hospital Affiliated to Tongji Medical College of Huazhong University of Science and Technology. Medical records of patients treated for upper limb lymphedema after breast cancer surgery between January 2021 and December 2023 were reviewed. Grouping was based on the treatment patients had received during routine clinical care. The study protocol was approved by the Ethics Committee of our hospital and followed the principles of the Declaration of Helsinki. Because this was a retrospective analysis, written informed consent was waived.

### 2.2. Patients

Women were included if they were 18 years of age or older, had previously undergone breast cancer surgery, and had unilateral upper limb lymphedema documented in the medical record on the basis of compatible clinical manifestations and physical examination. For retrospective case identification, records were also required to contain bilateral arm circumference measurements at the predefined sites used in this study, with a greater circumference on the affected side supporting the diagnosis. No single prespecified interlimb circumference cutoff (e.g., ≥2 cm) or formal lymphedema staging system was uniformly documented in the available records and therefore was not used as a mandatory inclusion criterion. Patients were additionally required to have complete baseline and posttreatment records and to have received either Sizi Powder external application combined with TDP therapy or conventional rehabilitation management during the study period.

Patients were not included if they had bilateral upper limb lymphedema, recurrent or metastatic breast cancer during the observation period, active skin infection, ulceration, or severe dermatitis of the affected limb, severe cardiac, hepatic, renal, or other systemic disease likely to interfere with treatment assessment, incomplete medical records, or additional intensive interventions that could substantially influence lymphedema outcomes.

### 2.3. Treatment

Patients in the combination group received Sizi Powder external application together with TDP therapy, in addition to routine rehabilitation care. Sizi Powder consisted of Ramulus Cinnamomi, *Asarum sieboldii*, *Sargentodoxa cuneata*, and Rhizoma Chuanxiong in equal proportions. For each treatment, the herbal powder was mixed with warm water to form a uniform paste, and an amount sufficient to cover the swollen area of the affected upper limb with a continuous layer was applied externally. The exact gram weight used per application was not consistently documented in the retrospective medical records and, therefore, could not be reliably reported. Each application session lasted 30 minutes and was performed once daily for 2 consecutive weeks.

TDP therapy was delivered using a therapeutic electromagnetic spectrum irradiation device. The lamp was positioned approximately 30 to 40 cm from the target area, with the distance adjusted when necessary according to local warmth and patient tolerance to avoid excessive heat. Each session lasted 30 minutes and was performed once daily for 2 consecutive weeks. The manufacturer and model of the TDP device were not consistently recorded in the retrospective medical records; therefore, the reproducible treatment parameters available for this analysis were treatment modality, irradiation distance, session duration, treatment frequency, and treatment course. Skin condition and local tolerance were monitored throughout treatment.

Patients in the control group received routine rehabilitation management alone, including limb elevation, skin care instruction, and guidance on upper limb functional exercise according to usual clinical practice. Supportive care was provided in both groups when needed, including advice on limb protection, avoidance of local injury or overuse, and encouragement of rehabilitation exercise.

Because Sizi Powder external application and TDP therapy were used together in the combination group, the present study was intended to assess the combined treatment strategy rather than the separate effect of either component.

### 2.4. Data collection

Clinical information was collected from the electronic medical record system using a standardized form. Baseline data included age, body mass index (BMI), affected side, type of surgery, axillary lymph node dissection, radiotherapy history, duration of lymphedema, baseline mean circumference difference, and baseline symptom scores. Information on treatment response, upper limb function, and adverse events was also recorded.

To reduce errors in data extraction, all records were checked independently by 2 investigators. When inconsistencies were found, the original charts were reviewed again until agreement was reached.

### 2.5. Outcome measures

The main outcomes were changes in upper limb swelling and symptom burden after treatment. Arm circumference was measured at 4 predefined sites: the wrist, 10 cm below the elbow, the elbow, and 10 cm above the elbow. At each site, the circumference difference was calculated by subtracting the value of the unaffected limb from that of the affected limb. The mean circumference difference across the 4 sites was also calculated and used as an overall measure of edema severity.

Symptoms including swelling, pain, heaviness, tightness, and numbness were assessed using routine clinical scoring. Each symptom was graded on a 4-point scale from 0 to 3, with higher scores indicating more severe symptoms. A total symptom score was obtained by summing the individual items.

Secondary outcomes included upper limb functional recovery, overall treatment response, and safety. Upper limb function was evaluated by shoulder flexion, shoulder abduction, shoulder external rotation, and the Disabilities of the Arm, Shoulder and Hand (DASH) score. A lower DASH score indicated better upper limb function.

Overall clinical efficacy was classified as markedly effective, effective, or ineffective according to improvement in edema and symptoms. Markedly effective was defined as a reduction in mean circumference difference of at least 50% together with marked symptom relief. Effective was defined as a reduction of 20% to 49% with partial symptom relief. A reduction of <20%, or no obvious symptomatic improvement, was regarded as ineffective. The overall response rate was calculated as the proportion of patients classified as either markedly effective or effective. For the multivariable logistic regression analysis, the dependent variable was dichotomized as clinical improvement (markedly effective or effective = 1) versus no clinical improvement (ineffective = 0).

Safety was assessed from treatment-related adverse events documented in the medical records. These included skin redness, itching, heat discomfort, worsening local pain, and other mild discomfort. Serious adverse events were defined as those requiring discontinuation of treatment, urgent medical intervention, or hospitalization.

### 2.6. Statistical analysis

Data analysis was performed using SPSS version 26.0 or R version 4.3.1. Continuous variables are presented as mean ± standard deviation, and categorical variables are presented as number and percentage. Distribution normality was examined with the Shapiro–Wilk test.

For continuous variables with a normal distribution, comparisons between groups were performed with the independent-samples *t* test, and changes before and after treatment within the same group were assessed with the paired *t* test. When variables were not normally distributed, the Mann–Whitney *U* test and Wilcoxon signed-rank test were used instead. Categorical variables were compared with the chi-square test or Fisher’s exact test.

To explore factors related to clinical improvement, multivariable logistic regression analysis was performed. The binary dependent variable was clinical improvement, defined as markedly effective or effective (coded as 1), versus ineffective (coded as 0). Treatment group, age, BMI, duration of lymphedema, radiotherapy history, and baseline edema severity were included as covariates. Odds ratios and 95% confidence intervals (CIs) were calculated. A 2-sided *P* value < .05 was considered statistically significant.

## 3. Results

### 3.1. Baseline characteristics

A total of 86 patients with upper limb lymphedema after breast cancer surgery were included in the final analysis, including 44 in the combination group and 42 in the control group. Baseline data are summarized in Table 1. No significant between-group differences were found in age, BMI, affected side, type of surgery, axillary lymph node dissection, radiotherapy history, duration of lymphedema, baseline mean arm circumference difference, or baseline symptom scores (all *P* > .05). These findings suggest that the 2 groups were broadly comparable at baseline.

**Table 1:** Baseline characteristics of the patients.

Variable	Combination group (n = 44)	Control group (n = 42)	*t/*χ^2^/*Z*	*P* value
Age (yr)	55.1 ± 7.6	53.8 ± 8.4	0.75	.454
BMI (kg/m^2^)	24.6 ± 3.2	25.0 ± 2.8	0.61	.544
Affected side (left/right)	25/19	20/22	0.63	.428
Mastectomy, n (%)	31 (70.5)	28 (66.7)	0.14	.709
Breast-conserving surgery, n (%)	13 (29.5)	14 (33.3)	0.14	.709
Axillary lymph node dissection, n (%)	29 (65.9)	30 (71.4)	0.30	.585
Radiotherapy history, n (%)	26 (59.1)	28 (66.7)	0.53	.469
Duration of lymphedema (mo)	11.2 ± 5.1	10.6 ± 4.8	0.56	.576
Baseline mean circumference difference (cm)	3.51 ± 0.88	3.32 ± 0.76	1.07	.288
Baseline swelling score	2.61 ± 0.56	2.47 ± 0.63	1.09	.280
Baseline pain score	2.21 ± 0.68	2.12 ± 0.59	0.66	.511
Baseline heaviness score	2.38 ± 0.61	2.29 ± 0.65	0.66	.512
Baseline tightness score	2.10 ± 0.72	2.03 ± 0.69	0.46	.648
Baseline numbness score	1.86 ± 0.73	1.79 ± 0.71	0.45	.651
Baseline total symptom score	11.16 ± 2.58	10.70 ± 2.47	0.84	.403

Data are presented as mean ± standard deviation or n (%), as appropriate.

BMI = body mass index.

### 3.2. Changes in arm circumference difference

Changes in arm circumference difference before and after treatment are shown in Table 2. Before treatment, circumference differences at the wrist, 10 cm below the elbow, the elbow, 10 cm above the elbow, and the overall mean were similar in the 2 groups. After treatment, swelling improved in both groups, but the reduction was greater in the combination group.

**Table 2:** Changes in arm circumference difference before and after treatment.

Measurement site	Combination group before treatment	Combination group after treatment	Control group before treatment	Control group after treatment	*P* value between groups after treatment
Wrist (cm)	2.24 ± 0.57	1.39 ± 0.46	2.08 ± 0.49	1.61 ± 0.50	.038
10 cm below elbow (cm)	3.31 ± 0.69	1.96 ± 0.58	3.17 ± 0.63	2.33 ± 0.61	.007
Elbow (cm)	3.72 ± 0.75	2.18 ± 0.63	3.54 ± 0.71	2.56 ± 0.67	.010
10 cm above elbow (cm)	4.76 ± 0.92	2.78 ± 0.77	4.48 ± 0.86	3.17 ± 0.79	.022
Mean circumference difference (cm)	3.51 ± 0.88	2.08 ± 0.56	3.32 ± 0.76	2.42 ± 0.60	.009

Values are expressed as mean ± standard deviation.

After treatment, the wrist circumference difference was lower in the combination group than in the control group (1.39 ± 0.46 cm vs 1.61 ± 0.50 cm, *P* = .038). The same pattern was seen 10 cm below the elbow (1.96 ± 0.58 cm vs 2.33 ± 0.61 cm, *P* = .007), at the elbow (2.18 ± 0.63 cm vs 2.56 ± 0.67 cm, *P* = .010), and 10 cm above the elbow (2.78 ± 0.77 cm vs 3.17 ± 0.79 cm, *P* = .022). The posttreatment mean circumference difference was also smaller in the combination group than in the control group (2.08 ± 0.56 cm vs 2.42 ± 0.60 cm, *P* = .009).

### 3.3. Changes in symptom scores

Symptom scores before and after treatment are presented in Table 3. At baseline, the 2 groups did not differ significantly in swelling, pain, heaviness, tightness, numbness, or total symptom score. After treatment, symptom severity decreased in both groups.

**Table 3:** Changes in symptom scores before and after treatment.

Outcome	Combination group before treatment	Combination group after treatment	Control group before treatment	Control group after treatment	*P* value between groups after treatment
Swelling score	2.61 ± 0.56	1.34 ± 0.51	2.47 ± 0.63	1.67 ± 0.55	.006
Pain score	2.21 ± 0.68	1.16 ± 0.52	2.12 ± 0.59	1.42 ± 0.57	.030
Heaviness score	2.38 ± 0.61	1.12 ± 0.49	2.29 ± 0.65	1.51 ± 0.53	.001
Tightness score	2.10 ± 0.72	1.28 ± 0.58	2.03 ± 0.69	1.43 ± 0.61	.248
Numbness score	1.86 ± 0.73	1.19 ± 0.56	1.79 ± 0.71	1.31 ± 0.60	.335
Total symptom score	11.16 ± 2.58	6.09 ± 1.95	10.70 ± 2.47	7.34 ± 2.08	.005

Each symptom score was graded on a clinical scale, with higher scores indicating more severe symptoms.

Compared with the control group, the combination group had lower posttreatment swelling scores (1.34 ± 0.51 vs 1.67 ± 0.55, *P* = .006), pain scores (1.16 ± 0.52 vs 1.42 ± 0.57, *P* = .030), and heaviness scores (1.12 ± 0.49 vs 1.51 ± 0.53, *P* = .001). The posttreatment total symptom score was also lower in the combination group (6.09 ± 1.95 vs 7.34 ± 2.08, *P* = .005). No significant between-group differences were observed in tightness or numbness after treatment.

### 3.4. Upper limb functional outcomes

Upper limb functional outcomes are listed in Table 4. Before treatment, shoulder flexion, shoulder abduction, shoulder external rotation, and DASH score were similar between the 2 groups. Functional improvement was observed after treatment in both groups, with greater improvement in some indicators in the combination group.

**Table 4:** Comparison of upper limb functional outcomes before and after treatment.

Functional indicator	Combination group before treatment	Combination group after treatment	Control group before treatment	Control group after treatment	*P* value between groups after treatment
Shoulder flexion (°)	130.8 ± 15.4	149.7 ± 12.1	132.1 ± 14.6	143.2 ± 13.0	.019
Shoulder abduction (°)	123.4 ± 17.1	146.2 ± 13.5	124.8 ± 16.3	138.4 ± 14.2	.011
Shoulder external rotation (°)	56.3 ± 8.7	66.7 ± 7.8	57.1 ± 8.0	63.8 ± 8.1	.098
DASH score	35.9 ± 8.9	23.4 ± 6.7	34.8 ± 8.1	27.2 ± 7.1	.014

Lower DASH scores indicate better upper limb function.

DASH = Disabilities of the Arm, Shoulder and Hand.

After treatment, shoulder flexion was higher in the combination group than in the control group (149.7 ± 12.1° vs 143.2 ± 13.0°, *P* = .019), and shoulder abduction showed a similar difference (146.2 ± 13.5° vs 138.4 ± 14.2°, *P* = .011). The posttreatment DASH score was lower in the combination group (23.4 ± 6.7 vs 27.2 ± 7.1, *P* = .014), indicating better upper limb function. Shoulder external rotation was also numerically higher in the combination group, although the difference did not reach statistical significance (66.7 ± 7.8° vs 63.8 ± 8.1°, *P* = .098).

### 3.5. Treatment response

Treatment response is shown in Table 5. In the combination group, 21 patients were classified as markedly effective, 18 as effective, and 5 as ineffective, corresponding to an overall response rate of 88.6%. In the control group, 14 patients were markedly effective, 17 were effective, and 11 were ineffective, giving an overall response rate of 73.8%. Although the response rate was higher in the combination group, the between-group difference was not statistically significant (*P* = .084).

**Table 5:** Comparison of treatment response between the 2 groups.

Response category	Combination group (n = 44)	Control group (n = 42)	χ^2^	*P* value
Markedly effective	21	14		
Effective	18	17		
Ineffective	5	11		
Overall response rate, n (%)	39 (88.6)	31 (73.8)	2.98	.084

Overall response rate = (markedly effective + effective)/total number of cases × 100%.

### 3.6. Safety outcomes

Adverse events are summarized in Table 6. Treatment-related adverse events were uncommon in both groups and were generally mild. In the combination group, 2 patients developed skin redness, 2 reported itching, and 1 experienced heat discomfort. In the control group, 1 patient had skin redness, 1 reported local pain aggravation, and 1 had another mild discomfort.

**Table 6:** Comparison of adverse events between the 2 groups.

Adverse event	Combination group (n = 44)	Control group (n = 42)	χ^2^/Fisher’s exact test	*P* value
Skin redness	2	1		
Itching	2	0		
Heat discomfort	1	0		
Local pain aggravation	0	1		
Other mild discomfort	0	1		
Total adverse events	5 (11.4)	3 (7.1)	0.47	.494

All adverse events were mild and self-limited. No serious treatment-related adverse events were observed in either group.

The total incidence of adverse events was 11.4% in the combination group and 7.1% in the control group, with no significant difference between groups (*P* = .494). All adverse events were self-limited, and no serious treatment-related adverse events occurred during the study period.

### 3.7. Multivariable analysis

The multivariable logistic regression results are shown in Table 7. After adjustment for age, BMI, duration of lymphedema, radiotherapy history, and baseline edema severity, combination treatment remained significantly associated with clinical improvement (odds ratio = 2.91, 95% CI: 1.03–8.22, *P* = .044). None of the other variables included in the model showed a significant association with clinical improvement.

**Table 7:** Multivariate logistic regression analysis of factors associated with clinical improvement.

Variable	OR	95% CI	*P* value
Combination treatment (yes vs no)	2.91	1.03–8.22	.044
Age	0.99	0.94–1.05	.733
BMI	0.93	0.79–1.10	.391
Duration of lymphedema	0.94	0.86–1.02	.128
Radiotherapy history	0.81	0.28–2.31	.689
Baseline edema severity	0.71	0.41–1.22	.216

Multivariate analysis was adjusted for age, BMI, duration of lymphedema, radiotherapy history, and baseline edema severity.

BMI = body mass index, CI = confidence interval, OR = odds ratio.

Although the overall response rate did not differ significantly in the unadjusted comparison, the adjusted analysis suggested that the combined intervention might still be associated with a higher likelihood of clinical improvement after accounting for baseline covariates.

## 4. Discussion

In this retrospective cohort study, patients treated with Sizi Powder external application combined with TDP therapy showed greater short-term improvement in several clinically relevant outcomes than those receiving routine rehabilitation care alone. The main between-group differences were observed in arm circumference difference, symptom burden, and selected indicators of upper limb function. Overall, the results suggest that this combined intervention may be useful as an adjunctive option in the rehabilitation of upper limb lymphedema after breast cancer surgery. These findings may be clinically relevant because BCRL is widely recognized as a multidimensional condition affecting limb volume, symptom burden, physical function, and quality of life.^[11-13]^

A notable result of this study was the greater reduction in arm circumference difference in the combination group. Persistent swelling is the core feature of BCRL and is closely related to long-term discomfort, reduced limb use, and impaired quality of life.^[14-17]^ Current rehabilitation practice has placed increasing emphasis on early recognition, surveillance, and supportive intervention to reduce progression to chronic or refractory lymphedema.^[18]^ In the present study, the combination group showed smaller posttreatment circumference differences at the wrist, forearm, elbow, and upper arm, together with lower swelling, pain, and heaviness scores and better recovery in selected functional outcomes. This pattern suggests that better edema control may have been accompanied by lower symptom burden and improved upper limb use in daily life. However, the magnitude of the between-group differences should be considered alongside statistical significance. The absolute posttreatment differences favoring the combination group were 0.22 cm at the wrist, 0.37 cm at 10 cm below the elbow, 0.38 cm at the elbow, 0.39 cm at 10 cm above the elbow, and 0.34 cm for the mean circumference difference. Similarly, shoulder flexion and abduction differed by 6.5° and 7.8°, respectively, and the DASH score differed by 3.8 points. These findings may represent an incremental short-term benefit, but this study did not prespecify minimal clinically important difference thresholds or include a patient global assessment; therefore, the clinical meaningfulness of these modest absolute differences cannot be established from *P* values alone. The absence of significant differences in tightness, numbness, and shoulder external rotation further supports a cautious interpretation and may indicate that the combined intervention has a more evident short-term effect on congestion-related symptoms than on chronic sensory complaints or all domains of upper limb function.^[19-21]^

These findings should also be interpreted in light of several methodological limitations. First, this was a single-center retrospective cohort study, and treatment allocation was based on routine clinical practice rather than randomization. As a result, selection bias, confounding by indication, and residual confounding cannot be excluded. Second, because Sizi Powder external application and TDP therapy were used together in the combination group, the independent contribution of each component could not be determined. Third, some practical intervention details, including the exact gram weight of herbal powder per application and the manufacturer/model of the TDP device, were not consistently documented in the retrospective records; although the preparation method and key treatment parameters available in the charts are now reported, this limits complete procedural replication. Fourth, some outcomes, particularly symptom scores, were based on routine clinical assessment rather than validated patient-reported instruments, which may have introduced measurement bias.^[22-24]^ In addition, multiple secondary comparisons were performed across circumference, symptom, and functional endpoints, raising the possibility of type I error, especially for outcomes with borderline statistical significance. These limitations are particularly relevant in the current evidence base for BCRL rehabilitation, where the quality and consistency of available guidance remain variable.^[25]^

Another issue that warrants caution is the difference between the unadjusted categorical efficacy analysis and the adjusted regression analysis. Although the overall response rate was numerically higher in the combination group, the between-group difference did not reach statistical significance. By contrast, multivariable logistic regression suggested that combination treatment was associated with clinical improvement after adjustment for selected baseline covariates. This discrepancy may reflect the effect of covariate adjustment, but it should still be interpreted carefully given the modest sample size and the relatively wide 95% CI (1.03–8.22), whose lower bound was close to the null value. Accordingly, the regression result should be viewed as supportive rather than definitive evidence of benefit. In addition, the short treatment duration and limited follow-up do not allow conclusions regarding durability of benefit, recurrence prevention, or long-term safety. Overall, the present study provides preliminary observational evidence that Sizi Powder external application combined with TDP therapy may serve as a useful adjunctive approach in postoperative rehabilitation for BCRL, but larger prospective, multicenter, and preferably randomized studies are still needed to confirm these findings and better define its role in clinical practice.

## 5. Conclusion

In this study, Sizi Powder external application combined with TDP therapy was associated with greater short-term improvement in limb swelling, symptom burden, and selected upper limb functional outcomes in patients with upper limb lymphedema after breast cancer surgery. The intervention was generally well tolerated in this cohort. Because this was a single-center retrospective study with a limited sample size and short follow-up, the findings should be interpreted cautiously. Larger prospective studies are needed to further clarify the clinical value of this combined approach.

## Author contributions

**Conceptualization:** Juan Zeng, Liwen Xu, Yi Zhang, Huili Xu.

**Data curation:** Juan Zeng, Liwen Xu, Yi Zhang, Huili Xu.

**Formal analysis:** Juan Zeng, Liwen Xu, Yi Zhang, Huili Xu.

**Funding acquisition:** Huili Xu.

**Investigation:** Huili Xu.

**Writing – original draft:** Huili Xu.

**Writing – review & editing:** Huili Xu.

## References

[1] Sung H Ferlay J Siegel RL. Global cancer statistics 2020: GLOBOCAN estimates of incidence and mortality worldwide for 36 Cancers in 185 Countries. CA Cancer J Clin. 2021;71:209–49. 10.3322/caac.21660 33538338

[2] McLaughlin SA Brunelle CL Taghian A. Breast cancer-related lymphedema: risk factors, screening, management, and the impact of locoregional treatment. J Clin Oncol. 2020;38:2341–50. 10.1200/JCO.19.02896 PMC7343436 32442064

[3] McLaughlin SA DeSnyder SM Klimberg S. Considerations for clinicians in the diagnosis, prevention, and treatment of breast cancer-related lymphedema: recommendations from a multidisciplinary expert ASBrS panel: part 1: definitions, assessments, education, and future directions. Ann Surg Oncol. 2017;24:2818–26. 10.1245/s10434-017-5982-4 28766232

[4] Executive Committee of the International Society of Lymphology. The diagnosis and treatment of peripheral lymphedema: 2023 consensus document of the International Society of Lymphology. Lymphology. 2023;56:133–51. 39207406

[5] Jørgensen MG Toyserkani NM Hansen FG Bygum A Sørensen JA. The impact of lymphedema on health-related quality of life up to 10 years after breast cancer treatment. npj Breast Cancer. 2021;7:70. 10.1038/s41523-021-00276-y PMC8169644 34075045

[6] Li L Yuan L Chen X. Current treatments for breast cancer-related lymphoedema: a systematic review. Asian Pac J Cancer Prev. 2016;17:4875–83. 10.22034/APJCP.2016.17.11.4875 PMC5454690 28030915

[7] Lin Y Yang Y Zhang X Li W Li H Mu D. Manual lymphatic drainage for breast cancer-related lymphedema: a systematic review and meta-analysis of randomized controlled trials. Clin Breast Cancer. 2022;22:e664–73. 10.1016/j.clbc.2022.01.013 35370085

[8] Hasenoehrl T Palma S Ramazanova D. Resistance exercise and breast cancer-related lymphedema-a systematic review update and meta-analysis. Support Care Cancer. 2020;28:3593–603. 10.1007/s00520-020-05521-x PMC7316683 32415386

[9] Hayes SC Stuiver MM Labrecque M. The effect of exercise for the prevention and treatment of cancer-related lymphedema: a systematic review and meta-analysis. Med Sci Sports Exerc. 2022;54:1389–99. 10.1249/MSS.0000000000002918 35320145

[10] Pereira ACPR Koifman RJ Bergmann A. Incidence and risk factors of lymphedema after breast cancer treatment: 10 years of follow-up. Breast. 2017;36:67–73. 10.1016/j.breast.2017.09.006 28992556

[11] Davies C Levenhagen K Ryans K Perdomo M Gilchrist L. Interventions for breast cancer-related lymphedema: clinical practice guideline from the academy of oncologic physical therapy of APTA. Phys Ther. 2020;100:1163–79. 10.1093/ptj/pzaa087 PMC7412854 32589208

[12] Rafn BS Christensen J Larsen A Bloomquist K. Prospective surveillance for breast cancer-related arm lymphedema: a systematic review and meta-analysis. j Clin Oncol. 2022;40:1009–26. 10.1200/JCO.21.01681 35077194

[13] Hendrickx AA Cornelissen AJM van der Hulst RRWJ Beugels J. Early referral for breast-cancer-related lymphedema: do we follow the evidence? A two-year prospective multicenter cohort study. Cancers (Basel). 2022;14:6016. 10.3390/cancers14236016 PMC9738967 36497495

[14] Wong HCY Rembielak A Montoya LEL. Multinational Association of Supportive Care in Cancer (MASCC) clinical practice guidance for the prevention of breast cancer-related arm lymphoedema (BCRAL): international Delphi consensus-based recommendations. EClinicalMedicine. 2024;68:102441. 10.1016/j.eclinm.2024.102441 PMC10850412 38333542

[15] Koelmeyer L Gaitatzis K Ridner SH. Implementing a prospective surveillance and early intervention model of care for breast cancer-related lymphedema into clinical practice: application of the RE-AIM framework. Support Care Cancer. 2021;29:1081–9. 10.1007/s00520-020-05597-5 PMC10979505 32613370

[16] Sharifi N Ahmad S. Breast cancer-related lymphedema: a critical review on recent progress. Surg Oncol. 2024;56:102124. 10.1016/j.suronc.2024.102124 39208532

[17] Shamoun S Ahmad M. Complete decongestive therapy effect on breast cancer related to lymphedema: a systemic review and meta-analysis of randomized controlled trials. Asian Pac J Cancer Prev. 2023;24:2225–38. 10.31557/APJCP.2023.24.7.2225 PMC10676504 37505751

[18] Gilchrist L Levenhagen K Davies CC Koehler L. Effectiveness of complete decongestive therapy for upper extremity breast cancer-related lymphedema: a review of systematic reviews. Med Oncol. 2024;41:297. 10.1007/s12032-024-02421-6 PMC11496316 39438358

[19] Jeffs E Ream E Taylor C Purushotham A Bick D. Evidence for the clinical effectiveness of decongestive lymphoedema treatment for breast cancer-related arm lymphoedema, a systematic review. Support Care Cancer. 2024;32:568. 10.1007/s00520-024-08759-x PMC11296965 39093326

[20] Dolgoy ND Al Onazi MM Parkinson JF. The appraisal of clinical practice guidelines for breast cancer-related lymphedema. Lymphat Res Biol. 2023;21:469–78. 10.1089/lrb.2022.0090 37037029

[21] Eaton LH Narkthong N Hulett JM. Psychosocial issues associated with breast cancer-related lymphedema: a literature review. Curr Breast Cancer Rep. 2020;12:216–24. 10.1007/s12609-020-00376-x PMC7445072 32864036

[22] Gursen C Dylke ES Moloney N. Self-reported signs and symptoms of secondary upper limb lymphoedema related to breast cancer treatment: systematic review. Eur J Cancer Care (Engl). 2021;30:e13440. 10.1111/ecc.13440 33733550

[23] Togawa K Ma H Smith AW. Self-reported symptoms of arm lymphedema and health-related quality of life among female breast cancer survivors. Sci Rep. 2021;11:10701. 10.1038/s41598-021-89055-0 PMC8139966 34021179

[24] Perdomo M Davies C Levenhagen K Ryans K Gilchrist L. Patient education for breast cancer-related lymphedema: a systematic review. J Cancer Surviv. 2023;17:384–98. 10.1007/s11764-022-01262-4 PMC9546750 36207626

[25] Shen A Lu Q Fu X. Risk factors of unilateral breast cancer-related lymphedema: an updated systematic review and meta-analysis of 84 cohort studies. Support Care Cancer. 2022;31:18. 10.1007/s00520-022-07508-2 36513801

